# Investigating the Impact of Pineapple–Whey Protein Fermentation Products on Cefixime-Induced Intestinal Flora Dysbiosis in Mice Using 16S Sequencing and Untargeted Metabolomics Techniques

**DOI:** 10.3390/foods13121927

**Published:** 2024-06-19

**Authors:** Jiawei Luo, Shan Xiao, Da Ma, Junhan Xiang, Bo Wang, Yanxue Cai, Jihui Wang

**Affiliations:** 1 School of Life and Health Technology, Dongguan University of Technology, Dongguan 523808, China; luojiawei615@163.com (J.L.); mada0471@163.com (D.M.); xiangjunhan2012@163.com (J.X.); bwang@dgut.edu.cn (B.W.); caiyanxue@dgut.edu.cn (Y.C.); wangjihui@dgut.edu.cn (J.W.); 2 Dongguan Prefabricated Food Innovation Development and Quality Control Key Laboratory, Dongguan 523808, China

**Keywords:** pineapple–whey protein fermentation product, cefixime, intestinal microbiota, metabolome

## Abstract

In our previous study, a new fermented food (PWF) created by utilizing pineapple by-products and whey proteins as a matrix via co-fermentation with lactic acid bacteria and yeast was developed, and, in the current study, we examined the impact of a pineapple–whey protein fermentation product on a cefixime-induced dysbiosis model in mice using 16S sequencing and untargeted metabolomics techniques. The results indicated that the pineapple–whey protein fermentation product played a positive role in restoring the intestinal flora. In this study, cefixime reduced the overall abundance of intestinal flora and decreased the relative abundance of probiotics in the gut, while also inhibiting amino acid metabolism. The addition of PWF normalized the intestinal flora to a steady state, significantly increasing the populations of *Weissella*, *Lactococcus*, *Faecalibaculum*, and *Bacteroides* acidophilus, while decreasing the numbers of *Akkermansia* and *Escherichia-Shigella*. Additionally, PWF modulated microbial metabolites, such as L-glutamate and L-threonine, and upregulated amino-acid-related metabolic pathways, including those involving glycine, serine, and threonine. In conclusion, PWF can alleviate intestinal flora dysbiosis and metabolic disturbances induced by antibiotic interventions. It is suggested that PWF could be a potential dietary strategy for patients with antibiotic-associated diarrhea.

## 1. Introduction

Recently, the World Health Organization highlighted the emergence of antibiotic resistance as a burgeoning global crisis. The inappropriate application and mismanagement of antibiotics comprises a major threat to public health [1]. In addition to obtaining highly effective therapeutic outcomes, the utilization of antibiotics has strong correlations with the prevalence of certain conditions, such as obesity, diabetes, asthma, and diarrhea [2]. These associations demonstrate the multifaceted impacts of applying antibiotics in medical treatments. Cefixime is classified as a third-generation cephalosporin antibiotic, and it is distinguished by structural enhancements compared with preceding cephalosporins. These modifications contribute to an expanded antibacterial spectrum, thereby making it efficacious against diverse bacterial strains. Cefixime is applied in the therapeutic management of a range of infections, including upper respiratory tract infections, lower respiratory tract infections, urinary tract infections, and skin and soft tissue infections [3]. The antibacterial mode of action for cefixime is based on the inhibition of bacterial cell wall synthesis, thereby restricting bacterial growth and reproduction. The inappropriate utilization of cefixime can potentially induce antibiotic-associated diarrhea and have detrimental effects on the intestinal microbiota [2]. Many studies have demonstrated that the consumption of fermented foods containing abundant probiotics and bioactive compounds can ameliorate disruption of the intestinal microbiota caused by antibiotic administration. This dietary intervention facilitates recovery of the human intestinal flora and enhances the host’s health [4].

The growing popularity of fermented foods in recent years can mainly be attributed to their discernible health benefits, particularly regarding human gastrointestinal health. During the fermentation of these foods, the microorganisms employed as fermentation starters contribute to the production of various advantageous components, including polyphenols, vitamins, and organic acids [4]. In addition, these microorganisms facilitate the breakdown of complex compounds in the fermentation matrix by transforming them into smaller nutrient molecules that are more readily absorbed by the body. Many empirical studies have shown that the microorganisms present in fermented foods can traverse the gastrointestinal tract to confer physiological benefits by competing with pathogenic bacteria and generating fermentation by-products that are conducive to immunomodulation [5]. Lactic acid bacteria comprise the main microorganisms used in the production of fermented foods, and they play key roles in the fermentation of dairy products, meat, fruits, and vegetables. Lactic acid bacteria are major probiotics within the human intestinal ecosystem, where they mainly colonize the duodenum through to the terminal ileum [6]. Empirical insights derived from a study conducted by Dimidi et al. [4] suggest that the ingestion of lactic acid bacteria and their metabolites can potentially elicit immune system stimulation, augment gastrointestinal function, and have favorable impacts on the blood glucose levels and cholesterol. Furthermore, Gryaznova et al. [6] showed that specific strains of lactic acid bacteria present in sauerkraut produce conjugated linoleic acid, which may have anti-cancer and anti-atherosclerotic properties. Yeasts comprise another category of microorganism with extensive applications in the fermentation of foods, where they impart distinctive organoleptic properties and enhance the nutritional contents [7]. A previous study demonstrated that Saccharomyces boulardii isolated from lychee may be efficacious in the treatment of irritable bowel syndrome due to the inhibition of gut motility through the upregulation of intestinal serotonin transporter proteins and modulation of the intestinal microbiota [8].

In our previous study, we formulated a novel fermented food by utilizing pineapple by-products and whey protein as the matrix for co-fermentation with indigenous strains of *Lactococcus lactis* LA5 and the yeast *Hanseniaspora opuntiae* SA2, which were both isolated from pineapple pulp. Our findings indicated that this newly developed fermented product was rich in probiotics, organic acids, phenols, active peptides, and other bioactive substances, as well as exhibiting favorable flavor and taste characteristics [9]. Furthermore, our in vitro simulated digestion and fermentation experiments demonstrated the beneficial effects of this fermented food on human intestinal flora, as well as promoting the production of short-chain fatty acids in the intestine, and functional analysis revealed that the product could regulate intestinal flora homeostasis. However, due to limitations inherent in in vitro experiments, further in vivo studies are necessary to validate these findings [10]. Consequently, in the present study, we investigated the impact of our pineapple by-product–whey protein fermented food (PWF) on the intestinal flora and metabolites in mice following cefixime intervention. The findings obtained in this study contribute to a more comprehensive understanding of the potential implications of fermented foods for human gastrointestinal health and function.

## 2. Materials and Methods

### 2.1. Sample Preparation and Collection

The samples were prepared as described in our previous study [9]. The pineapples (*Ananas comosus* L.) were thoroughly cleaned, and both the peel and flesh were removed to isolate the pineapple cores. These cores were pulped using a high-speed blender and subsequently mixed with distilled water at a homogenate: water mass ratio of 6:4. Whey protein (2.6%, *w*/*w*) was incorporated into the mixture, before adjusting the pH to 5.0. Subsequently, the substrate mixture was sterilized in a water bath at 65 °C for 30 min. After revitalization and activation of lactic acid bacteria and yeasts for fermentation, the starter culture strain was inoculated into the fermentation substrate at a volume ratio of 6:1 for lactic acid bacteria (*Lactococcus lactis* LA5) and yeast (*Hanseniaspora opuntiae* SA2), with a 1.6 mL/100 g (mL/w) inoculum of lactic acid bacteria. The concentration was set at 1 × 10^6^ CFU/mL for both lactic acid bacteria and yeast. The mixture was incubated at 37.5 °C for 26 h in the absence of light. After fermentation, the samples were freeze-dried for 48 h to obtain a powdered product and then stored at −80 °C until subsequent experiments.

### 2.2. Animal Treatments

**Figure 1:**
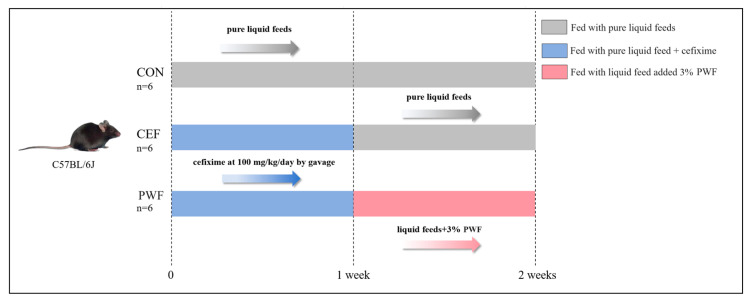
Timeline of the experiment. CON, control group; CEF, model group; PWF, intervention group.

C57BL/6J mice (6 weeks old, male, license number SCXK2020-0005) were procured from Skibbes Biotechnology Co (Zhengzhou, China). Animal experiments were approved and performed based on the ethical and animal experimental guidelines of Beijing HFK BioScience Co., Ltd. (Beijing, China), approval number IACUC-20230901. All animal experiments strictly adhered to the principles outlined in the Guide for the Care and Use of Laboratory Animals. Mice were housed in a regulated environment with a temperature maintained at 23–25 °C, relative humidity set at 50–55%, and a 12 h light:12 h dark cycle. After acclimatization for one week, the mice were randomly allocated to three groups (n = 6 per group), which were housed separately, comprising the control group (CON), cefixime group (CEF), and PWF intervention group (PWF). The mice were provided with standard liquid chow (TP4020C). Mice in the CON group were given regular pure liquid chow for two weeks. Mice in the CEF group received 100 mg/kg/day of cefixime by gavage in addition to pure liquid chow during the first week, followed by the same diet as the CON group during the second week. Mice in the PWF group received the same treatment as those in the CEF group during the first week, and they were subsequently fed liquid chow containing 3% PWF during the second week [3,11]. The specific experimental flow is shown in Figure 1. The daily food intake by mice was documented, and their body weights were systematically recorded at intervals of three days. Fecal specimens were systematically collected on days 7 and 14. Fecal samples collected promptly after excretion were transferred to sterile tubes, frozen rapidly using liquid nitrogen, and subsequently stored at −80 °C. Following the experimental period of 14 days, mice were euthanized via cervical dislocation. The colons were cleansed with phosphate-buffered saline to ascertain their lengths. The colons were then immersed in 10% formalin solution for subsequent histopathological analysis.

### 2.3. Histomorphological Analysis of Colons

The colonic tissues were fixed, embedded in paraffin, sectioned, and stained with hematoxylin and eosin (H&E), according to conventional procedures. The histomorphology of the colon was then observed through microscopy.

### 2.4. Analysis of Gut Microflora

Microbial community samples were obtained from the feces of each group of mice using a QIAamp Fast DNA Faecal Kit. The V4 region of the 16S rRNA gene was subsequently amplified using the primers 515F (GTG CCA GCMGCC GCG GTAA) and 806R (GGA CTA CHVGGG TWT CTAAT). Following amplification, the DNA concentration was quantified using a microplate reader (Tecan, Infinite 200 Pro, Bern, Switzerland). Fragment size analysis was performed via agarose gel electrophoresis (1.5% *w*/*v*), with an expected size of 600 bp for the 16S region.

Next-generation sequencing was conducted using the Illumina MiSeq Platform (Illumina, San Diego, CA, USA) from Azenta Life Sciences Inc. (South Plainfield, NJ, USA). The sequencing process involved automated cluster generation and 250/300 paired-end sequencing with dual reads, according to the manufacturer’s instructions. Prior to sequencing, a filtering step was applied to eliminate sequences containing ambiguous bases (“N”) and sequences with lengths below 200 bp. Quality filtering was performed to remove low-quality sequences and chimeric sequences. The resulting sequences were clustered into operational taxonomic units (OTUs) using the VSEARCH clustering method (version 1.9.6) with a sequence similarity threshold of 97%.

Silva (version 138) was used as the reference database for taxonomic analysis of the 16S rRNA gene. The Ribosomal Database Program (RDP) classifier, based on the Bayesian algorithm for OTU species taxonomy analysis, was employed to assign taxonomic classifications to representative sequences. This comprehensive analysis process was conducted to examine the microbial community compositions at different taxonomic levels for each sample, providing detailed insights into the diversity and relative abundance of microbial taxa [12].

### 2.5. Analysis of the Metabolome

Fecal samples weighing approximately 100 mg were extracted using a cold solvent comprising 80% (*v*/*v*) methanol and 0.1% (*v*/*v*) formic acid. After extraction for 1 min, the samples were centrifuged at 15,000× *g* and 4 °C for 15 min. The resultant supernatant was collected and diluted to a final methanol concentration of 53% (*v*/*v*), and 100 μL/sample was extracted. This extract was mixed with 400 μL of extraction solution (MeOH:ACN, 1:1 (*v*/*v*)), which contained deuterated internal standards. The mixture was vortexed for 30 s, sonicated for 10 min in a water bath at 4 °C, and incubated for 1 h at −40 °C to induce protein precipitation [13]. Subsequently, the samples were centrifuged at 12,000 rpm (relative centrifugal force = 13,800× *g*, R = 8.6 cm) and 4 °C for 15 min. The supernatant was then transferred to a new glass vial for subsequent analysis. A quality control sample was prepared by combining equal aliquots of the supernatant from all samples. Liquid chromatography–tandem mass spectrometry (LC-MS/MS) analyses were conducted using a Ultra-High Performance Liquid Chromatography (UHPLC) system (Vanquish, Thermo Fisher Scientific, Waltham, MA, USA) coupled with a Waters ACQUITY UPLC BEH Amide column (2.1 mm × 50 mm, 1.7 μm) and an Orbitrap Exploris 120 mass spectrometer (Orbitrap MS, Thermo). The mobile phases consisted of 25 mmol/L ammonium acetate and 25 mmol/L ammonia hydroxide in water (pH = 9.75, A) and acetonitrile (B). The auto-sampler temperature was maintained at 4 °C, and the injection volume was 2 μL. The Orbitrap Exploris 120 mass spectrometer operated in the information-dependent acquisition mode to continuously evaluate full-scan mass spectra. The electrospray ionization source conditions included a sheath gas flow rate of 50 Arb, an aux gas flow rate of 15 Arb, a capillary temperature of 320 °C, a full MS resolution of 60,000, an MS/MS resolution of 15,000, collision energy set at SNCE 20/30/40, and a spray voltage of 3.8 kV (positive) or −3.4 kV (negative).

Raw data were converted into the mzXML format using ProteoWizard and then processed with an in-house program developed in R utilizing XCMS for peak detection, extraction, alignment, and integration. Following these steps, an in-house MS2 database was utilized for metabolite annotation employing a cutoff threshold of 0.3. Metabolite identification levels were assigned based on a previous study [14]. The subsequent analysis was based on data derived from reliable matches, which were established through MS/MS comparisons with a standard database. This rigorous approach ensured that the identified metabolites were accurately characterized, allowing for detailed and reliable interpretation of the metabolomic data.

### 2.6. Statistical Analysis

Statistical analyses were conducted using R software R 3.6.0+ and SPSS 17.0 software. Principal component analysis (PCA) was conducted using the princomp function in R. Linear discriminant effect size analyses were conducted using the Kruskal–Wallis test incorporating all adversarial strategies for multicategory analysis. Differences in the alpha diversity index, relative abundance of species, and relative abundance of genes were compared using one-way analysis of variance in SPSS. Subsequently, Duncan’s multiple range test was applied to assess differences between groups. A significance threshold of *p* < 0.05 was applied for all analyses.

## 3. Results

### 3.1. Subsection

**Figure 2:**
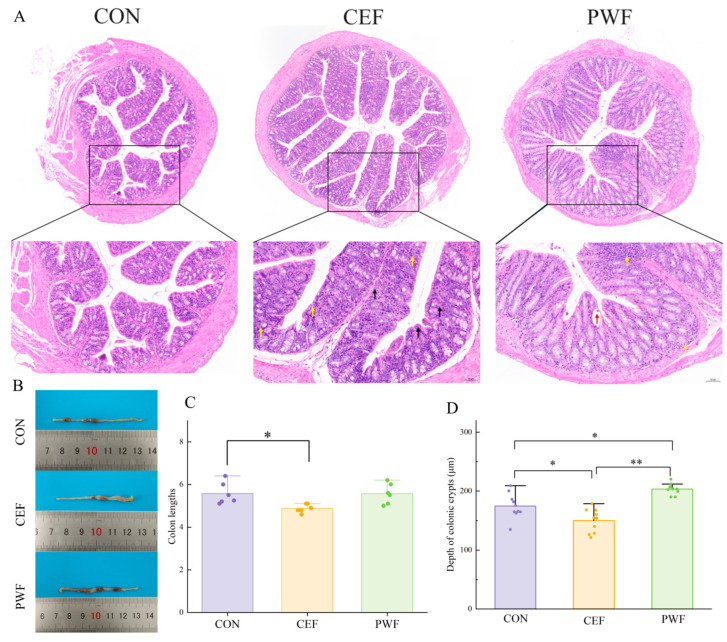
Colon characteristics of mice in CON, CEF, and PWF groups. (**A**) Histomorphology of the colon after hematoxylin and eosin staining. (**B**,**C**) Colon lengths in mice. (**D**) Depths of crypts in mice colons. Significant differences are indicated by * *p* < 0.05, ** *p* < 0.01. Yellow arrows indicate inflammatory cell infiltration; red arrows indicate visible abscesses; black arrows indicate cell necrosis.

The colon lengths in mice are illustrated in Figure 2B. Clearly, the mice in the CEF group tended to have shorter colons, whereas the colon length in the PWF group did not differ significantly from that in the CON group. As shown in Figure 2A, the morphology of the colon tissue structure was mainly normal in the CON group. The epithelial cells within the mucosal layer were characterized by tight and orderly arrangements without detachment. The crypts exhibited structural integrity and a well-organized arrangement. The submucosal layer showed no signs of edema, and evident inflammatory cell infiltration in the tissues was absent. In the CEF group, the colonic tissues exhibited structural aberrations. The epithelial cells within the mucosal layer were tightly arranged in the observed field, and they did not show signs of detachment. Individual crypts appeared slightly swollen, and the submucosal layer did not show indications of edema. The tissue was characterized by substantial infiltration of inflammatory cells, a notable prevalence of necrotic cells, fragmented cell nuclei, and deeply stained fixative. In the PWF group, minor abnormalities were observed in the colonic tissue structure. The epithelial cells within the mucosal layer were tightly arranged in the observed field, with no evidence of detachment. Abscesses were noted in individual crypts, and edema was not observed in the submucosal layer. In addition, a small amount of inflammatory cell infiltration was observed in the tissue. We also observed and measured the crypt fossa depth in mice subjected to different treatments. The images indicated substantial reductions in the colonic crypt depth among mice in the CEF group compared with the CON group. In contrast, mice in the PWF group were characterized by a marked increase in the colonic crypt depth compared with those in the CON group (Figure 2C). These findings suggest that the consumption of PWF increased the colonic crypt depth in mice.

### 3.2. Effects of PWF on Body Weight and Food Intake by Mice

**Figure 3:**
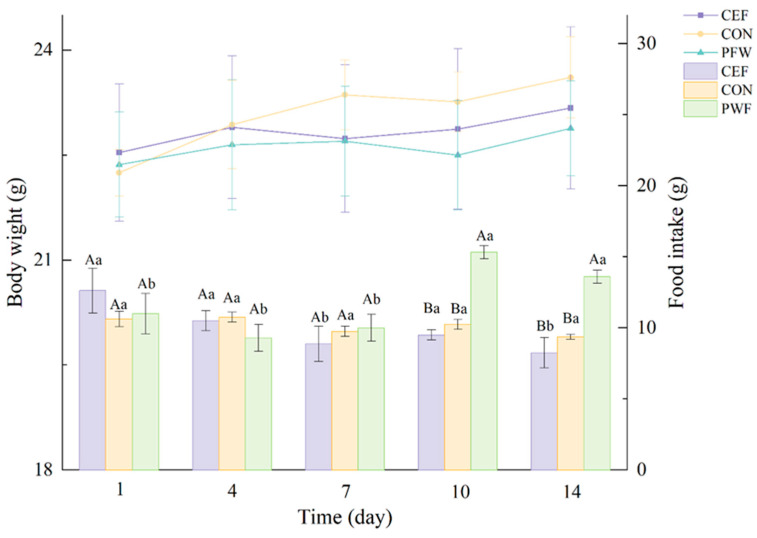
Changes in body weight and food intake of mice from different groups during the experiment. Different capital letters (A,B) indicate significant differences between groups on the same day, and different lowercase letters (a,b) indicate significant differences between groups on different days.

The changes in the average body weight and food intake in the three groups of mice are shown in Figure 3. Mice in the PWF group exhibited increased food consumption following PWF treatment compared with the other two groups. However, no significant differences were observed in the average body weight gains among the three groups of mice throughout the experiment. These findings indicate that PWF could increase the food intake by mice, but without causing discernible changes in body weight.

### 3.3. Effects of PWF on Microbial Community Richness and Diversity

**Figure 4:**
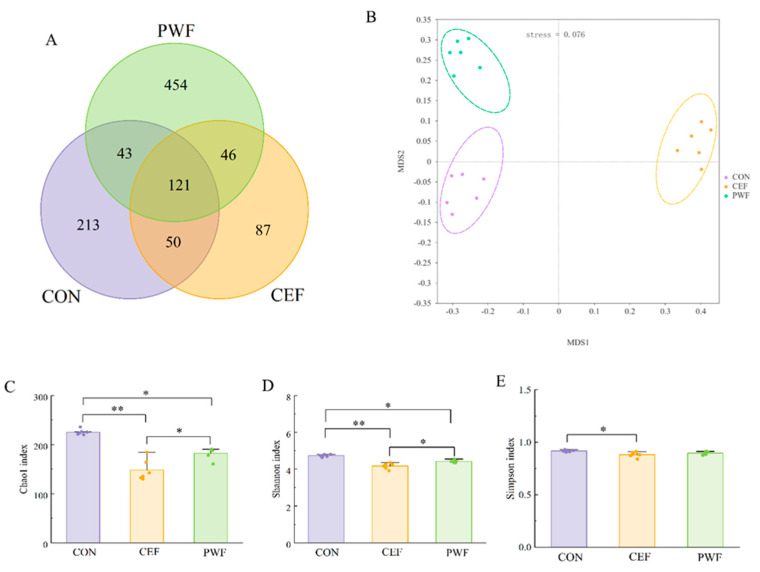
Microbial community diversity analysis of mice colons from different groups. (**A**) Venn diagram showing shared and unique OTUs among different groups. (**B**) Non-Metric Multidimensional Scaling of different groups. (**C**–**E**) correspond to different groups in terms of the Chao1 index, Shannon index, and Simpson index, respectively. Significant differences are indicated by * *p* < 0.05, ** *p* < 0.01.

All gut microbes were classified into 1014 OTUs with a 97% similarity threshold based on sequence data. Figure 4A shows that 121 of the 1014 OTUs were present in all groups, where the CEF group had the lowest total number of OTUs, and thus the least diverse gut microbial community. In contrast, the PWF group had a significantly higher total number of OTUs compared with the CEF group, and thus the highest gut microbial diversity. Therefore, incorporating PWF into the diet helped to restore the gut microbial diversity in mice following cefixime administration. To ensure the reliability of our results, we assessed the Chao1 index, Shannon index, and Simpson index for each group of microbial communities. As shown in Figure 4C–E, significant differences in the Chao1 index and Simpson index were observed between the CON and CEF groups, thereby indicating that cefixime treatment markedly decreased the richness and diversity of gut microbe species in mice, which did not recover to normal levels even after 14 days. In contrast, no significant differences were found in the Simpson index between the CON and PWF groups, thereby supporting the efficacy of PWF at enhancing the gut microbial diversity after antibiotic treatment. To comprehensively analyze the overall structure of the gut microbial community, we conducted Non-Metric Multidimensional Scaling (NMDS) to evaluate the beta diversity of the gut microbial communities in different groups of mice. Through NMDS analysis, the species information within the samples is represented in multidimensional space as points, with the distance between points reflecting the degree of difference between samples. This allows for a more comprehensive assessment of inter- and intra-group differences among samples. As shown in Figure 4B, the stress value of the NMDS model was less than 0.2, indicating its ability to accurately reflect the degree of difference between samples. The clusters in the CEF group were clearly separated compared with the CON group, thereby demonstrating significant changes in the gut microbial community in mice following cefixime treatment. In contrast, the microbial community structure in the PWF group closely resembled that in the CON group, and thus the PWF actively contributed to recovery from the changes in the gut microbial community structure induced by antibiotics.

### 3.4. Effects of PWF on Composition of the Gut Microbial Community

**Figure 5:**
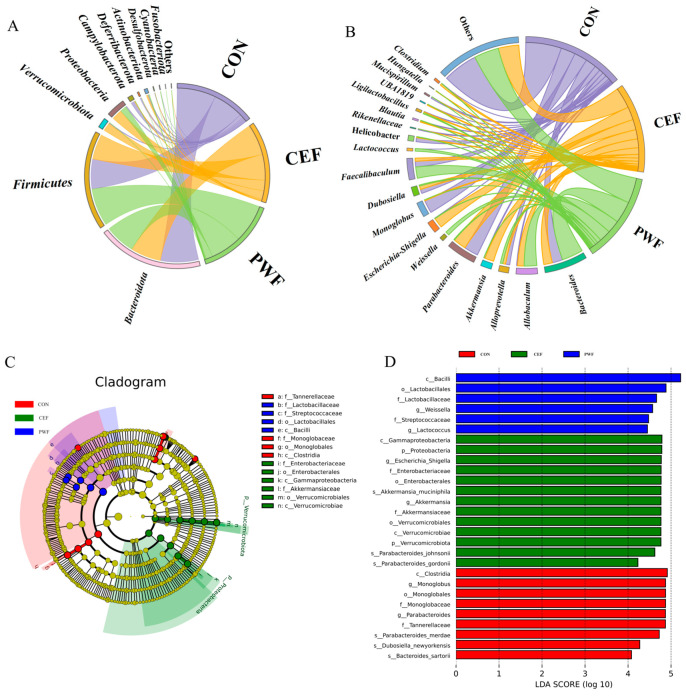
Composition of the gut microbial community in mice colons from different groups. (**A**) Composition of microbial guts during the experiment. (**B**) Composition of genus-level microbes during the experiment. (**C**,**D**) LDA-based cladogram and distribution histogram, respectively.

The gut microbial community compositions were measured in mice. As shown in Figure 5A, Firmicutes and Bacteroidetes were the dominant phyla in all groups. Compared with the CON group, the abundances of *Verrucomicrobiota* and *Proteobacteria* were significantly higher in the CEF group, whereas the abundances of *Firmicutes* and *Bacteroidetes* were lower. In contrast, in the PWF group, the relative abundances of *Bacteroidota*, *Campylobacterota*, and *Deferribacterota* were significantly lower, and the relative abundances of *Firmicutes* and *Proteobacteria* were higher. These findings suggest that the gut microbial compositions differed at the phylum level among samples from the three groups.

The gut microbial community compositions were also examined in detail at the genus level in different samples. Figure 5B shows the top 20 bacterial genera with the highest relative abundances. Compared with the CON group, the relative abundances of *Allobaculum*, *Akkermansia*, *Escherichia–Shigella*, and *Clostridium* were significantly higher in the CEF group, whereas the relative abundances of *Monoglobus* and *Mucispirillum* were significantly lower. In contrast, compared with the CEF group, the relative abundances of *Bacteroides* and *Faecalibaculum* were significantly higher in the PWF group, whereas the relative abundances of *Parabacteroides* and *Escherichia–Shigella* were significantly lower. Thus, significant changes in the intestinal bacterial genera occurred after feeding with PWF following cefixime treatment. It should be noted that PWF significantly increased the relative abundances of probiotic bacteria, such as *Weissella* and *Lactococcus,* compared with the CON group, which indicates that PWF can potentially increase the proportion of probiotic bacteria within the gut microbial community.

We conducted statistical analyses by using linear discriminant effect size analysis to further investigate biomarkers that indicated significant differences in the relative abundances of gut microbes between different groups of mice. Microorganisms that characterized the gut flora in different experimental groups were assessed based on the effect size of linear discriminant analysis (LDA), and the results are presented in Figure 5C,D. The LDA histograms (Figure 5D) indicated statistically significant differences in the microbial communities. At the phylum level, the CEF group was enriched for *Verrucomicrobiota* and *Proteobacteria* (LDA > 4), but no community enrichment was observed at the phylum level in the other groups. At the genus level, the CON group was enriched for *Parabacteroides* and *Monoglobus* (LDA > 4). In contrast, the CEF group was enriched for *Escherichia–Shigella* from *Proteobacteria* and *Akkermansia* (LDA > 4). In addition, *Weissella*, *Lactococcus*, and *Faecalibaculum* were enriched in the phylum *Firmicutes* in the PWF group (LDA > 4) compared with the CEF group. These microorganisms were identified as potential indicator biomarkers for each group.

### 3.5. Analysis of Relative Abundances of Potential Biomarkers in Each Group

**Figure 6:**
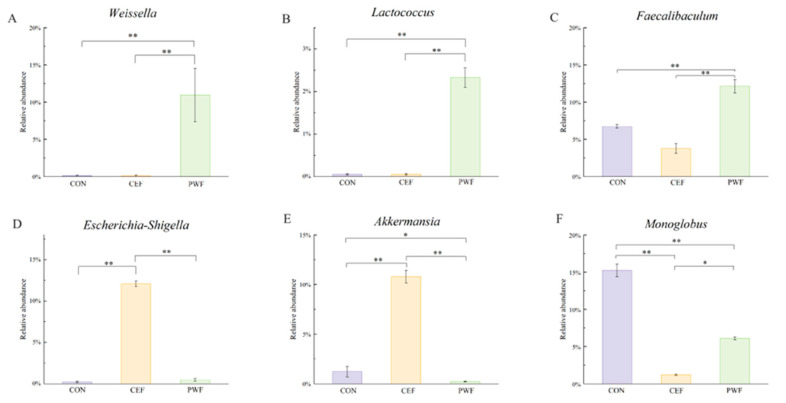
Relative abundances of (**A**) *Weissella*, (**B**) *Lactococcus*, (**C**) *Faecalibaculum*, (**D**) *Escherichia–Shigella*, (**E**) *Akkermansia*, and (**F**) *Monoglobus* from different groups. Significant differences are indicated by * *p* < 0.05, ** *p* < 0.01.

To further analyze the impact of PWF on gut microorganisms, we assessed the significant differences in the relative abundances of potential biomarkers in each experimental group, and the results are shown in Figure 6. The absence of Weissella and Lactococcus from the CON and CEF groups was particularly noteworthy, whereas the relative abundances of these microorganisms were significantly higher in the PWF group, as shown in Figure 6A,B. Similarly, the relative abundance of Faecalibaculum was 1.81 and 3.22 times higher in the PWF group compared with the CON and CEF groups, respectively (Figure 6C). Intriguingly, no statistically significant differences were observed in the relative abundances of Escherichia–Shigella between the PWF and CON groups. However, the relative abundance of Escherichia–Shigella was 27.68 times lower in the PWF group compared with the CEF group (Figure 6D). Furthermore, a congruent pattern was observed in terms of the relative abundance of Akkermansia, where its abundance was significantly lower in the PWF group compared to the CEF group (Figure 6E). Moreover, the relative abundance of Monoglobus was lower in both the CEF and PWF groups compared with the CON group, as shown in Figure 6F.

### 3.6. Effects of PWF on Fecal Metabolomics in Mice

**Figure 7:**
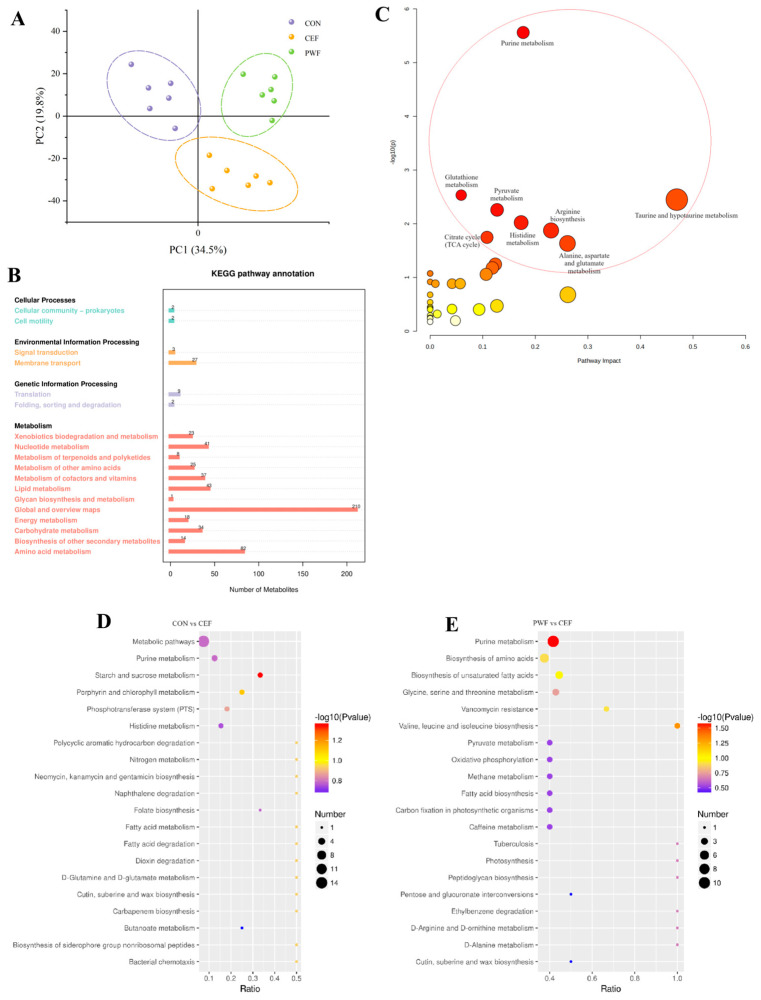
Metabolomic analysis of mice feces from different groups. (**A**) Principal component analysis based on the three experimental groups. (**B**) Number of enriched KEGG pathways. (**C**) Pathway enrichment map. (**D**,**E**) Bubble plots of KEGG pathways enriched in different groups.

We also investigated the fecal metabolome in mice. Using principal component analysis, we systematically examined the compositions of metabolites within fecal samples derived from the three groups of mice, as shown in Figure 7A. The results clearly indicated that there were substantial variations in the fecal metabolites among the three groups, and thus PWF affected the metabolic pathways and metabolites in the gut microbiota following antibiotic treatment.

In order to further assess the underlying pathways affected, we conducted enrichment analysis for fecal metabolites using the Kyoto Encyclopedia of Genes and Genomes (KEGG) pathway database. At the primary pathway level, mouse fecal metabolites were mainly enriched in the metabolism pathway. At the secondary pathway level, enriched metabolites were identified in Global and Overview Maps, amino acid metabolism, and Lipid Metabolism pathways (Figure 7B).

To elucidate the impacts of PWF on the metabolic pathways in the microbial communities in mouse feces, differential analysis of metabolic pathways was conducted among the different groups. Figure 7C,D show that the most significant differences in metabolic pathways were found between the CON group and CEF group. The main differentially regulated pathways included purine metabolism, the phosphotransferase system (PTS), histidine metabolism, folate biosynthesis, butanoate metabolism, and starch and sucrose metabolism. Furthermore, the main differentially regulated metabolic pathways between the PWF and CEF groups comprised purine metabolism, biosynthesis of amino acids, biosynthesis of unsaturated fatty acids, glycine, serine, and threonine metabolism, and valine, leucine, and isoleucine biosynthesis.

### 3.7. Correlations between Metabolites of Interest within Divergent Metabolic Pathways and Potential Microorganism Markers

We determined significant metabolites linked to different metabolic pathways in the three experimental groups. In addition, Pearson’s correlation coefficients were calculated to assess the associations between these metabolites and potential marker microorganisms in each experimental group.

**Figure 8:**
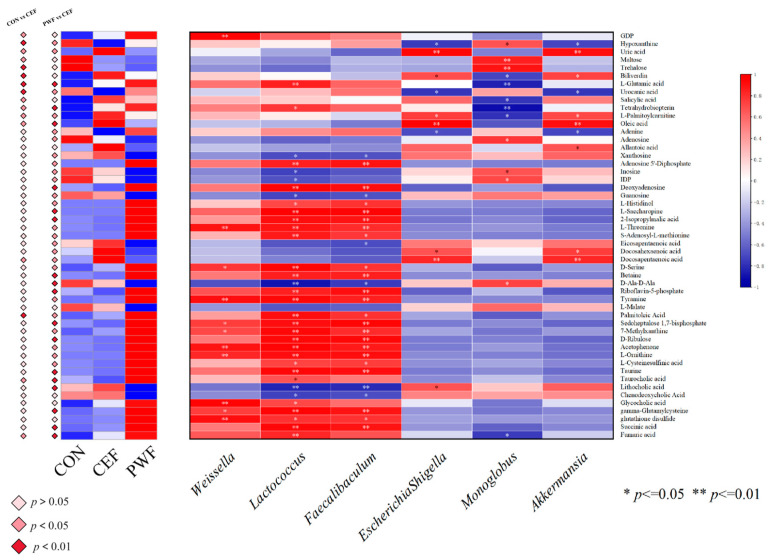
Differential metabolites and Spearman’s correlation coefficients between potential marker microorganisms and metabolites in three groups.

We screened 50 metabolites, and most had associations with primary metabolic pathways. In particular, 12 metabolites were identified in the Nucleotide metabolism pathway comprising guanosine 5’-diphosphate (GDP), hypoxanthine, uric acid, adenine, adenosine, allantoic acid, xanthosine, adenosine 5-diphosphate, inosine, Inosine 5’-diphosphate (IDP), deoxyadenosine, and guanosine. We also identified 19 metabolites in the amino acid metabolism pathway, comprising L-glutamic acid, urocanic acid, L-histidinol, L-saccharopine, 2-isopropylmalic acid, L-threonine, and S-adenosyl-L-methionine. Furthermore, metabolites linked with the carbohydrate metabolism pathway comprised maltose, trehalose, L-glutamic acid, 2-isopropylmalic acid, L-malate, D-ribulose, succinic acid, and fumaric acid. The metabolites linked with the lipid metabolism pathway comprised L-palmitoylcarnitine, oleic acid, eicosapentaenoic acid, docosahexaenoic acid, docosapentaenoic acid, palmitoleic acid, taurocholic acid, lithocholic acid, chenodeoxycholic acid, glycocholic acid, and 10 others. In addition, six metabolites were identified in the energy metabolism pathway, comprising L-glutamic acid, adenosine 5-diphosphate, riboflavin-5-phosphate, tyramine, L-malate, and sedoheptulose 1,7-bisphosphate. These metabolites differed significantly among the three experimental groups, as shown in Figure 8.

Figure 8 shows the correlations between members of the gut microbial communities and metabolites, which suggests that these potential microbial markers may have affected the overall composition of the gut flora, metabolism, and host health by modulating specific metabolites within metabolic pathways. In particular, Weissella had positive correlations with GDP, L-threonine, D-serine, riboflavin-5-phosphate, tyramine, sedoheptulose 1,7-bisphosphate, 7-methylxanthine, acetophenone, L-ornithine, glycocholic acid, gamma-glutamylcysteine, glutathione disulfide, and fumaric acid, and a negative correlation with D-Ala-D-Ala. Similarly, Lactococcus had significant correlations with several metabolites, such as positive associations with 27 substances, including L-glutamic acid, tetrahydrobiopterin, adenosine 5-diphosphate, deoxyadenosine, and L-histidinol, and negative correlations with 9 substances, including xanthosine, inosine, and guanosine. Faecalibaculum was highly negatively correlated with xanthosine, guanosine, eicosapentaenoic acid, D-Ala-D-Ala, lithocholic acid, and chenodeoxycholic acid, and Faecalibaculum was highly positively correlated with 23 metabolites, including L-threonine and D-serine. Escherichia–Shigella had positive correlations with uric acid, biliverdin, L-palmitoylcarnitine, oleic acid, docosahexaenoic acid, docosapentaenoic acid, and lithocholic acid, and negative correlations with hypoxanthine, urocanic acid, and adenine. Monoglobus had positive correlations with hypoxanthine, maltose, trehalose, adenosine, inosine, IDP, and D-Ala-D-Ala, and negative correlations with biliverdin, L-glutamic acid, salicylic acid, tetrahydrobiopterin, L-palmitoylcarnitine, and fumaric acid. Akkermansia had positive correlations with hypoxanthine, urocanic acid, adenine, deoxyadenosine, and riboflavin-5-phosphate, and negative correlations with uric acid, biliverdin, L-palmitoylcarnitine, oleic acid, allantoic acid, docosahexaenoic acid, and docosapentaenoic acid.

## 4. Discussion

The commensal microbiota found in the intestinal tract are intricately connected with the health status of the host organism. Disruption of homeostasis within the gut microbial community can greatly affect the physiological functionality to hinder the symbiotic relationship with the host organism [15]. Many studies have demonstrated the deleterious impacts of antibiotic administration on the intestinal microbiota [5,16]. These studies have shown the capacities of antibiotics to modify the compositional dynamics of the intestinal microbial community to reduce the abundances of probiotic bacteria and decrease the relative abundances of pathogenic bacteria, but also to influence the overall metabolic profile of the intestinal flora [17,18]. These perturbations make it difficult to restore the microbial community structure to a state of healthy equilibrium, with adverse consequences for the host [2]. In the present study, we found differences in the Chao1 and Simpson’s indexes for intestinal flora in the antibiotic-treated CEF group and CON group, as illustrated in Figure 4. These findings strongly indicate that the administration of antibiotics greatly reduced the richness and diversity of the intestinal flora, and similar findings were obtained by Ramirez et al. [2]. Antibiotic interventions can reduce the overall diversity of gut microbial species, which may lead to metabolic alterations and facilitate the development of bacterial antibiotic resistance. In addition, examination of the structural composition of the gut flora detected a substantial decrease in the abundance of *Bacteroidota* and a notable increase in the abundance of *Proteobacteria* due to antibiotic treatment. At the genus level, the antibiotic treatment led to significant increases in the relative abundances of detrimental gut microorganisms, particularly *Escherichia–Shigella* (Figure 5A,B). Xu et al. [19] also observed that extended antibiotic treatment led to reductions in the richness and diversity of the intestinal flora, but with increases in the relative abundances of deleterious genera. Moreover, Jo et al. [20] demonstrated that prolonged antibiotic treatment was associated with great reductions in the abundances of common beneficial species within the gut flora, and we also found a lack of probiotics in the CEF group in the present study. Hence, it is important to identify efficacious functional food additives in order to mitigate antibiotic-induced intestinal dysbiosis, preserve homeostasis of the intestinal flora, and increase the abundances of probiotic microorganisms.

In our previous studies, we demonstrated the proficient transit of microorganisms from probiotic-enriched PWF through the digestive system and their successful colonization of the intestinal tract. Moreover, PWF was shown to enhance the diversity of the gut microflora, increase the relative abundance of probiotics within the intestinal tract, and inhibit specific pathogenic flora [10]. However, the influence of PWF on the recovery of gut microbiota following antibiotic treatment was not elucidated. Thus, in the present study, we systematically evaluated the modulatory effect of orally administered PWF on antibiotic-induced dysbiosis of the gut flora. Our findings indicated that the diversity and abundance of the intestinal flora were significantly increased by PWF supplementation. Compared with the CEF group, reductions in the relative abundances of *Firmicutes*, *Proteobacteria*, *Escherichia–Shigella*, *Akkermansia*, and *Parabacteroides* were found in the PWF group, but notable increases were found in the relative abundances of *Weissella*, *Lactococcus*, *Faecalibaculum*, and *Bacteroides* (Figure 5A,B).

*Firmicutes* and *Bacteroidetes* are the main microbial phyla within the murine gastrointestinal tract, and changes in their relative abundances have been implicated in the pathogenesis of various disorders, including depression, anxiety, inflammatory bowel disease, and obesity [21]. In the present study, the relative abundances of *Bacteroidetes* did not differ significantly between the PWF and CEF groups. However, the relative abundance of *Firmicutes* was higher in the PWF group, possibly due to the increased relative abundances of probiotic bacteria at the genus level, such as *Weissella* and *Faecalibaculum*, within the murine intestinal tract. Li et al. identified an association between an increase in the abundance of *Firmicutes* in the intestinal microbiota and the development of obesity in host individuals [3]. In the present study, the mice in the PWF group were characterized by a significant increase in their daily food intake after PWF treatment. Interestingly, despite their greater food consumption, the body weights of mice in the PWF group did not differ significantly compared with those in the CON and CEF groups, possibly due to the enriched dietary fiber content present in PWF. The correlation between the abundance of *Firmicutes* and dietary fiber was also highlighted by Nobel et al. [22], who showed that an elevated abundance of *Firmicutes* in the gut was associated with increased dietary fiber intake. Importantly, their findings suggest that low dietary fiber intake might contribute to weight gain and obesity, whereas a high dietary fiber intake could potentially enhance the production of short-chain fatty acids in the colon to improve systemic insulin sensitivity [22].

*Lactococcus*, *Weissella*, and *Faecalibaculum* were identified as potential marker microorganisms in the PWF group, and their relative abundances were significantly higher compared with those in the CEF group. *Lactococcus* was applied as the fermentation strain for PWF production, and our previous study demonstrated that *Lactococcus* bacteria within PWF could effectively withstand gastrointestinal digestion to successfully colonize the colon [10]. *Lactococcus* is widely utilized in food fermentation and dairy production, and it has attracted attention because of its potential immune-modulatory effects. Saleena et al. [23] reported that *Lactococcus* may exert a modulatory influence on the host immune system, possibly by activating immune cells or modulating immune-related gene expression to positively affect the immune function. In addition, *Lactococcus* plays roles in certain pathways, such as fatty acid synthesis in the gut, to produce secondary metabolites with inhibitory effects on the proliferation of deleterious microorganisms within the gastrointestinal tract [24]. In our previous empirical study, we identified *Weissella* as a major microbial genus in PWF [9]. *Weissella* is frequently found in fermented foods and fermentation processes, and it has attracted attention because of its potential probiotic attributes [25,26]. Lakra et al. elucidated the antimicrobial and antioxidant capabilities of *Weissella* isolated from fermented dough and demonstrated its probiotic potential [27]. In addition, Xie et al. [28] demonstrated that the extracellular polysaccharides produced by *Weissella* exhibit prebiotic properties with implications for mitigating obesity. In the present study, *Weissella* had positive correlations with metabolites, such as succinic acid, fumaric acid, and riboflavin-5-phosphate, which are associated with carbohydrate and energy metabolism. These correlations suggest a potential role for *Weissella* in promoting carbohydrate and energy metabolism. In particular, increased glucose catabolism and facilitation of the carbohydrate metabolism pathway may lead to enhanced energy provision. The concurrent upregulation of energy metabolism could potentially lead to increased energy expenditure and fat oxidation, thereby suggesting that *Weissella* may be a possible preventive agent against obesity. *Faecalibaculum* is a key constituent of the human intestinal microbiota, where it comprises 5–15% of the total bacterial population detected in fecal samples from healthy individuals. This bacterium is significant as one of the principal generators of butyric acid, which is acknowledged because of its anti-inflammatory properties and roles in maintaining the enzyme activities of bacteria and protecting the digestive system against intestinal pathogens [29]. In this study, we found that *Faecalibaculum* had a strong positive correlation with palmitoleic acid. In a previous study, Ding et al. demonstrated that palmitoleic acid promoted fatty acid production, which suggests a potential relationship between *Faecalibaculum* and palmitoleic acid in the complex metabolic processes related to fatty acid metabolism [30].

*Akkermansia* is a major colonizer of the intestinal mucosal layer, and it has attracted attention because of its evident physiological benefits both in vitro and in vivo. *Akkermansia* is regarded as a potential next-generation probiotic, and its efficacy as a probiotic is based on maintaining its normal abundance [31]. In the present study, the relative abundance of *Akkermansia* was significantly higher in the CEF group than the CON and PWF groups, as also reported by Yuan et al. [13], who showed that *Akkermansia* adheres to the intestinal mucus layer. However, it should be noted that excessive mucosal attachment by *Akkermansia* may potentially compromise the integrity of the mucus layer to affect the functionality of the intestinal barrier. As shown in Figure 3, mice in the CEF group were characterized by aberrant intestinal tissue structure, with a lower number of crypts and greatly reduced colonic crypt depth, possibly due to the increased abundance of *Akkermansia*. In contrast, the abundance of *Akkermansia* was more normal in the PWF group compared with the CEF group, and the associated intestinal tissue structure appeared to be normal. The crypts remained intact, and the submucosal layer did not have edematous characteristics. These observations suggest that PWF supplementation could potentially ameliorate the intestinal flora dysbiosis induced by antibiotic treatment to help restore a more balanced microbiota composition.

*Monoglobus* adheres to the mucosa, and it has been implicated in the preservation of the intestinal mucosal layer and other physiological functions. Morais et al. [32] observed an increase in the relative abundance of *Monoglobus* in the intestinal tract following the consumption of chia seed powder. In addition, a strong positive correlation was found between *Monoglobus* and the crypt depth in the colon, which agrees with the findings obtained in the present study [32]. We found that the relative abundance of *Monoglobus* was significantly lower in the CEF group compared with both the CON and PWF groups. Moreover, the crypt depth differed significantly in the CEF group compared with the CON and PWF groups. The crypt depth is a significant parameter as an indicator of colonic health because it contributes to increasing the intestinal surface area to enhance the absorption of nutrients and preserve normal physiological functions. Moreover, an increase in the crypt depth is important for maintaining the integrity of the intestinal mucosal layer by acting as a strong protective barrier against the ingress of deleterious substances into the body [33]. The intestinal structure and crypt depth were significantly better in the PWF and CON groups compared with the CEF group in the present study, thereby suggesting a beneficial role for PWF in facilitating recovery of the intestinal flora and maintaining the health of the intestinal structure.

Alterations in the intestinal flora, particularly microbes with specific functionalities, can induce subsequent shifts in the biometabolic pathways related to all intestinal microflora [3]. In the present study, the metabolites originating from the intestinal flora were mainly enriched in the metabolism pathway. Differential pathway analysis showed that antibiotic treatment induced alterations in the biometabolic pathways of the intestinal flora. In particular, significant changes in the metabolites maltose and trehalose associated with starch and sucrose metabolism were observed in the CEF group compared with the CON group. The substantial decreases in the contents of both metabolites in the CEF group indicated downregulation of the starch and sucrose metabolism pathway. Wu et al. [34] reported that the downregulation of starch and sucrose metabolism may attenuate the production of short-chain fatty acids in the intestine, which are crucial products of gut microbial metabolism with profound implications for the host’s intestinal health and immune function. In addition, starch and sucrose are crucial energy sources, and their metabolism involves a diverse array of enzymes and regulatory factors. Thus, the downregulation of this pathway may weaken the activity of relevant enzymes to affect the absorption of essential nutrients. Moreover, the eicosapentaenoic acid, docosahexaenoic acid, and docosapentaenoic acid contents were significantly higher in the CEF group after antibiotic treatment, thereby suggesting the upregulated biosynthesis of unsaturated fatty acids. Li et al. [35] proposed that the upregulation of this pathway may lead to the excessive synthesis of unsaturated fatty acids and potentially contribute to perturbed lipid metabolism, which has been linked with the development of cardiovascular diseases, such as those involving high cholesterol and triglyceride levels. Furthermore, the upregulation of unsaturated fatty acids that act as regulators in inflammatory responses may exacerbate inflammatory reactions to potentially influence the onset and progression of inflammatory diseases. Hypoxanthine is a metabolite linked to purine metabolism, and its level was lower in the CEF group, thereby indicating downregulation of purine metabolism in the CEF group of mice compared with the CON group. Hu et al. demonstrated an association between purine metabolism and the maintenance of intestinal barrier integrity, and changes in purine metabolism can potentially influence the synthesis of crucial substances that might compromise the integrity of the intestinal mucosa [36]. These findings agree with the results obtained in the present study.

**Figure 9:**
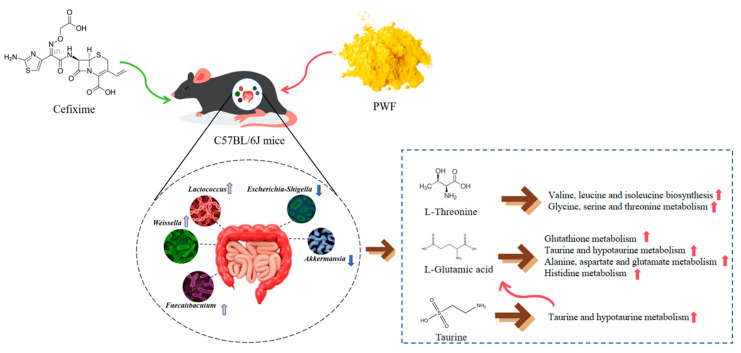
Restoration of cefixime-induced gut microflora dysbiosis by PWF in vivo model.

However, PWF ameliorated the perturbed biometabolic pathways due to the dysregulatory effects of antibiotics on the gut flora composition of mice. Metabolites associated with amino acid metabolism were significantly enriched in the PWF group compared with the CEF group. In particular, levels of the metabolites 2-isopropylmalic acid and L-threonine linked to valine, leucine, and isoleucine biosynthesis were significantly higher in the PWF group compared with the CEF group, which indicated the upregulation of this metabolic pathway. Valine, leucine, and isoleucine are essential branched-chain amino acids that must be consumed in the diet because they cannot be synthesized in the body. Some studies suggest that these amino acids possess anti-inflammatory properties and contribute to regulating the inflammatory response to sustain the host’s health status [37,38]. In addition, the levels of D-serine and betaine were higher in the PWF group than the CEF group. D-serine, betaine, and L-threonine are important metabolites associated with the glycine, serine, and threonine metabolism pathway, and thus this pathway was apparently upregulated in the PWF group. Glycine is a key precursor in the synthesis of proteins, nucleic acids, and lipids. Glycine is initially utilized as an energy source by the colonic flora and subsequently metabolized by the liver into glycogen and uric acid, which are organic compounds that contribute to the body’s energy reserves. Serine is a non-essential amino acid used as a nutrient by intestinal bacteria, and it undergoes conversion through gastrointestinal fermentation into another organic compound that is absorbed by the body to provide additional energy. Moreover, serine is a precursor for the synthesis of proteins and nucleic acids, and thus it has crucial roles in cell proliferation and survival. Similarly, threonine plays a pivotal role in the synthesis of adenosine triphosphate, which is vital for cellular metabolism. Furthermore, threonine contributes to the formation of collagen and elastin, which are essential components of connective tissue [39,40]. In the present study, L-glutamic acid was identified as a key metabolic product, where it had significant associations with some enriched differential metabolic pathways. The L-glutamic acid levels were significantly higher in the PWF group compared with the CEF group, possibly due to the significant increase in the taurine concentration in the PWF group compared with the CEF group. Both L-glutamic acid and taurine had strong associations with the taurine and hypotaurine metabolism pathway. Furthermore, taurine can potentially be converted into L-glutamic acid through the complex taurine and hypotaurine metabolism pathway. Upregulation of the taurine and hypotaurine metabolism pathway has the capacity to modulate the functionality of immune cells and influence inflammatory responses, thereby contributing to regulatory effects on the host immune system, as well as promoting the production of short-chain fatty acids by the intestinal flora [41]. L-glutamic acid is involved in the synthesis and metabolism of diverse amino acids [41]. In the present study, the succinic acid level was significantly higher in the PWF group compared with the CEF group. It should be noted that both succinic acid and L-glutamic acid had associations with the alanine, aspartate, and glutamate metabolism pathway. Furthermore, an analogous upregulation trend was observed for L-histidinol and urocanic acid in the PWF group, and these metabolites as well as L-glutamic acid are important for the histidine metabolism pathway. These findings suggest that PWF treatment enhanced the production of several amino acids by the intestinal flora to positively contribute to host recovery. Similarly, Al Mughram et al. [37] observed that upregulation of the alanine, aspartate, and glutamate metabolism pathway could lead to the concomitant upregulation of glutathione metabolism, where this correlation was attributed to the role of alanine as a glutathione metabolite and a precursor in glutathione metabolism. The upregulation of glutathione metabolism had beneficial effects on antioxidative processes, detoxification mechanisms, and immunomodulation within the intestinal tract, which are consistent with the results obtained in the present study (Figure 9).

## 5. Conclusions

In the present study, the administration of an antibiotic led to profound changes in both the composition and functionality of the gut microbiota. However, PWF administration helped to increase the abundance and diversity of the gut microbiota following antibiotic treatment to effectively rectify the dysbiotic state of the gut microbiota. The modulatory effects of PWF on the gut microbiota were demonstrated by the increases in the relative abundances of beneficial probiotic taxa, including *Faecalibaculum*, *Weissella*, and *Lactococcus*, as well as preventing the overgrowth of potentially deleterious microorganisms, such as *Akkermansia* and *Escherichia–Shigella.*

Furthermore, PWF regulated key metabolites, such as L-threonine and L-glutamic acid, to influence crucial metabolic pathways, particularly pathways related to amino acid metabolism in the gut microflora to modulate the microbial community and associated metabolites, with the potential to facilitate host recovery.

Consequently, PWF is a promising functional food additive for mitigating the adverse effects of antibiotic cefixime by generating a favorable microbial milieu in the gastrointestinal tract. However, this study only explored the beneficial impact of PWF on dysbiosis induced by the specific antibiotic cefixime. It remains uncertain whether PWF can ameliorate the effects of other antibiotics on the intestinal flora, necessitating further experiments to investigate its broader applicability.

## Data Availability

The original contributions presented in the study are included in the article, further inquiries can be directed to the corresponding author.
